# Fluid dipping technology of chimpanzees in Comoé National Park, Ivory Coast

**DOI:** 10.1002/ajp.22628

**Published:** 2016-12-21

**Authors:** Juan Lapuente, Thurston C. Hicks, K. Eduard Linsenmair

**Affiliations:** ^1^ Animal Ecology and Tropical Biology, Biozentrum Universität Würzburg Tierökologie und Tropenbiologie (Zoologie III) Würzburg Germany; ^2^ The Faculty of Artes Liberales University of Warsaw Warsaw Poland; ^3^ Department of Primatology Max Planck Institute for Evolutionary Anthropology Leipzig Germany

**Keywords:** brush tipped tool, fluid dipping, tool use, water‐acquisition, Western chimpanzee (*Pan troglodytes verus*)

## Abstract

Over a 6 month period during the dry season, from the end of October 2014 to the beginning of May 2015, we studied tool use behavior of previously unstudied and non‐habituated savanna chimpanzees (*Pan troglodytes verus*) living in the Comoé National Park, Ivory Coast (CI). We analyzed all the stick tools and leaf‐sponges found that the chimpanzees used to forage for ants, termites, honey, and water. We found a particular behavior to be widespread across different chimpanzee communities in the park, namely, dipping for water from tree holes using sticks with especially long brush‐tip modifications, using camera traps, we recorded adults, juveniles, and infants of three communities displaying this behavior. We compared water dipping and honey dipping tools used by Comoé chimpanzees and found significant differences in the total length, diameter, and brush length of the different types of fluid‐dipping tools used. We found that water dipping tools had consistently longer and thicker brush‐tips than honey dipping tools. Although this behavior was observed only during the late dry season, the chimpanzees always had alternative water sources available, like pools and rivers, in which they drank without the use of a tool. It remains unclear whether the use of a tool increases efficient access to water. This is the first time that water dipping behavior with sticks has been found as a widespread and well‐established behavior across different age and sex classes and communities, suggesting the possibility of cultural transmission. It is crucial that we conserve this population of chimpanzees, not only because they may represent the second largest population in the country, but also because of their unique behavioral repertoire.

## Abstract 2

Chimpanzees in Comoé National Park use specialized stick tools to collect water from tree holes (WDTs). WDTs are significantly different from tools used to gather honey. The use of WDTs is widespread across neighboring communities in the park.

## 1 INTRODUCTION

Chimpanzee tool use has been the subject of systematic study since the 1960s (Goodall, 1968; Jones & Sabater, 1969; Nishida, 1973; Sabater Pi, 1974), revealing regional and local variations that have been argued to be similar to culturally transmitted practices in humans (Boesch & Tomasello, 1998; McGrew, 1992; Whiten et al., 1999, 2001; Wrangham, de Waal, & McGrew, 1994). Western chimpanzees have been found to have several tool use behaviors unknown in other chimpanzee populations, such as nut cracking, studied since the 1970s both in the Tai Forest, CI (Boesch & Boesch, 1990), and Bossou, Guinea (Matsuzawa, Humle, & Sugiyama, 2011), and the hunting of *Galago* spp. with spears in Fongoli, Senegal (Pruetz & Bertolani, 2007). More recently, Kühl et al. (2016) documented accumulative stone throwing in Western chimpanzee communities of Guinea‐Bissau, Guinea‐Conackry, Liberia, and Ivory Coast, where chimpanzees habitually bang and throw rocks against trees, or toss them into tree cavities, resulting in conspicuous stone accumulations at these sites. Fluid dipping behavior is defined as the use of a probe to extract fluids such as honey or water from cavities (Boesch & Boesch, 1990; Nishida et al. 2010; Whiten et al., 1999, 2001; Wrangham et al., 1994). Honey dipping with probes is a quite widespread behavior, found in West African sites in Ivory Coast (Boesch & Boesch, 1990), Senegal (Bermejo, Illera, & Sabater Pí, 1989) and Nigeria (Dutton & Chapman, 2014; Sommer, Buba, Jesus, & Pascual‐Garrido, 2012), many Central African sites (Hicks, Fouts, & Fouts, 2005; Sanz & Morgan, 2007, 2009), as well as East Africa (Goodall, 1968; Gruber, Muller, Strimling, Wrangham, & Zuberbühler, 2009; Watts, 2008). The use of probes to dip for water, however, has only been described in Kibale (Wrangham et al., 1994) with the occasional use of chewed stems of Aframomum, and more recently, in a playing context by immature Mahale chimpanzees (Matsusaka et al., 2006). In both cases, the behavior was not habitual or customary, but only present in a small number of individuals.

The use of sponges, made by folding or chewing leaves to collect water from ponds, streams, or holes in trees has been reported in many chimpanzee communities across Africa (Goodall, 1968; Matsusaka et al., 2006; Sugiyama, 1995; Whiten et al., 1999, 2001, Sanz & Morgan, 2007). Matsuzawa (1991, cited in Sugiyama, 1995) found, however, that the Western chimpanzees of Bossou occasionally produced a local variant of leaf sponging behavior, by dropping leaf‐sponges into tree holes too narrow to access by hand and then retrieving them with the help of sticks. This behavior, defined as “push‐pull,” has been seen only rarely at other sites (Matsusaka et al., 2006; Sugiyama, 1995; Tonooka, 2001).

Chimpanzees inhabiting savanna‐woodland mosaics and dry environments have been studied more closely in East Africa (Goodall, 1968; Hunt and McGrew, 2002; Nishida, 1973; Wrangham et al., 1994), while in West Africa, the only area where long term studies in this type of habitat have been carried out is SE Senegal (i.e., Fongoli and Mount Assirik) (McGrew, Baldwin, & Tutin, 1981; Pruetz & Bertolani, 2007; Tutin, McGrew, & Baldwin, 1983) and more recently in Gashaka‐Gumti, Nigeria, for *P. t. ellioti* (Dutton & Chapman, 2014). As a result of behavioral adaptations to dry habitats and extreme seasonality, savanna chimpanzees are reported to exhibit seasonal changes in their movements across home‐ranges, which are linked to water availability, migrating and concentrating around the scarce water sources during the peak of dry season (Baldwin, McGrew, & Tutin, 1982; Duvall, 2000, 2008; Hunt & McGrew, 2002; Pruetz & Bertolani, 2009; Tutin et al., 1983). Other chimpanzee behaviors linked to water in some of these areas include digging wells in order to filter water (Galat, Galat‐Luong, & Nizinski 2008; Hunt & McGrew, 2002) as well as bathing in ponds, probably to cool down (Pruetz & Bertolani, 2009). In Tongo, DRC, which is an especially dry environment located on top of a lava flow with no access to surface water, chimpanzees drink from tree holes using moss sponges and dig up and consume tubers as probable alternative source of water (Lanjouw, 2002). Although water is a limiting factor for chimpanzees, they are able to adapt to living in marginal habitats as long as they have access to some water source on a daily basis (Duvall, 2000; Pruetz & Bertolani, 2009).

Even though chimpanzees in the Ivory Coast (CI) have been studied since the 1970s in the Taï Forest and surrounding areas (Boesch & Boesch, 1990, Hoppe‐Dominik, 1991), in the rest of the country, in particular in the Comoé National Park located in the northeast, research on chimpanzee has been limited to a few general censuses (Campbell, Kuehl, N'Goran‐Kouamé, & Boesch, 2008; Hoppe‐Dominik, 1991; Marchesi, Marchesi, Fruth, & Boesch, 1995) and occasional observations (Fischer & Gross, 1999), nevertheless, feeding ecology of other primates, such as baboon (*Papio anubis*), has been thoroughly studied in Comoé (Kunz & Linsenmair, 2010). Prior to our work, nothing was known about the distribution, ecology and behavior of the Comoé chimpanzees, which inhabit a mosaic of differing savanna types, gallery forest and forest islands. It has been argued that savanna‐woodland mosaics represent one of the environments in which human ancestors appear to have lived during much of the first 6 million years of their evolution (Cerling et al., 2011; Henry et al., 2012). Human ancestors probably found the same tool materials and faced problems in these habitats similar to those modern chimpanzees face, such as water and food scarcity. The technological solutions developed by wild chimpanzees to confront the challenges of this environment can provide a model for early technological adaptations of hominins (Wynn, Hernandez‐Aguilar, Marchant, & McGrew, 2011). Chimpanzee populations are threatened, due to factors such as poaching and habitat destruction (Campbell et al., 2008; Hicks et al., 2010) and with the extinction of local communities we may lose critical information on behavioral variability and technological adaptations present in great apes. Comoé chimpanzees live in a changing environment, where water passes from being abundant during the rainy season to be scarce and unevenly distributed during the dry season. Considering that chimpanzees in other study sites have been found to modify their behavior to adapt to dry environments (Baldwin et al., 1982; Duvall, 2000, 2008; Hunt & McGrew, 2002; Lanjouw, 2002; Pruetz & Bertolani, 2009; Tutin et al., 1983), we expect that Comoé chimpanzees developed behaviors to confront their dry savanna‐woodland environment. Accordingly, we test the following hypotheses:

1. Assuming that chimpanzees modify their behavior to confront savanna‐woodland dry conditions, we expect to find evidence of adaptive tool‐use behavior produced by Comoé chimpanzees.
2. Assuming that tool technology in wild chimpanzees is spread by social transmission, we expect water dipping behavior to be common and widespread across the studied chimpanzee communities.
3. Assuming that tools used by chimpanzees are specific to a particular task, we test the hypothesis that tools produced by Comoé chimpanzees to collect water are different in dimensions and structure from those made to dip for and harvest honey.
4. Given the evidence that the chimpanzees produce tools with long brush‐tips specifically to dip for water, we expect a direct correlation between brush length and amount of water absorbed.
5. Considering that water is a less dense fluid than honey and longer brush tips will be needed to absorb enough water per dip, we expect water dipping tool brush‐tips to be longer than honey dipping tool ones.

## 2 METHODS

### 2.1 Study area

**Figure 1:**
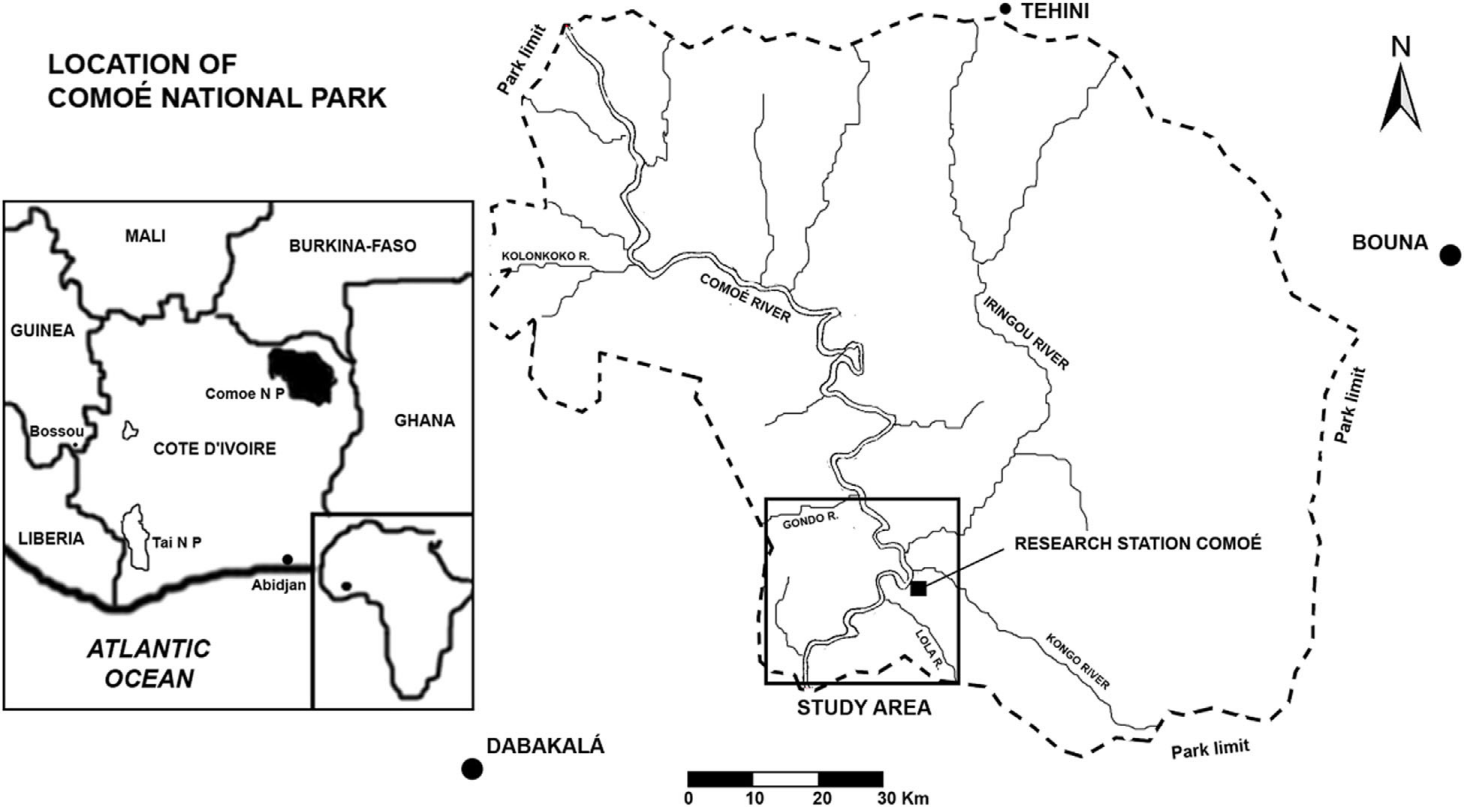
Location of Comoé National Park, detailing the placement of the study area

Comoé National Park is a protected area covering 11,500 km^2^ located in the NE of Ivory Coast (Figure 1). Savanna habitats cover 91% of the park, with the remaining area consisting of gallery forest and forest islands, which serve as the main chimpanzee habitat (Mühlenberg, Galat‐Luong, Poilecot, Steinhauer‐Burkart, & Kühn, 1990).

Comoé's tropical climate is warm and dry, with a mean temperature of 27°C and an average annual precipitation oscillating around 1090 mm (Hennenberg, 2005), based on data from two meteorological stations located 74 and 109 km, respectively, from the center of the study area. Our own measurements of rainfall at the Comoé Research Station, averaged 1010 mm during the period between 1993 and 2000 (Fischer, Gross, & Linsenmair, 2002). The dry season lasts for 6 months from November to April, with virtually no precipitation in December and January, and scattered rainstorms during the rest of the dry season. Total average precipitation during the dry season is around 100 mm (Fischer et al., 2002).

The Comoé River divides the park from N to S, and most of the park's known chimpanzee population resides to the west of the river. A network of tributaries crisscrosses the park, but most become completely dry or only retain small puddles of water during the dry season.

In addition to chimpanzee, we confirmed the presence of nine of the 14 species of primates that Fischer et al. documented in 2002, including baboon (*P. anubis*), patas monkey (*Erythrocebus patas*), white naped mangabey (*Cercocebus lunulatus*), Lowe's monkey (*Cercopithecus lowei*), lesser spot‐nose monkey (*Cercopithecus petaurista*), green monkey (*Chlorocebus sabaeus*), Demidoff's galago (*Galagoides demidoff*), Senegal galago (*Galago senegalensis*), and common potto (*Perodicticus potto*). Regarding large predators at the site, the presence of lion (*Panthera leo*) has not been confirmed after 2002, but leopards (*Panthera pardus*) and spotted hyenas (*Crocuta crocuta*) are common (Henschel et al., 2010, personal observations). In the Comoé River and its main tributaries, West African crocodyle, *Crocodylus suchus* and slender‐snouted crocodyle *C. cataphractos* are still present [personal observations].

We chose a 900 km^2^ study area in the SW sector of the park because it covers most of the range in which chimpanzees had been detected in previous surveys (Campbell et al., 2008; Hoppe‐Dominik, 1991; Marchesi et al., 1995), and also because Würzburg University has established the well‐equipped Research Station Comoé (RSC) in the core of this study area, considerably improving logistics and sample analysis in the field. In order to facilitate behavioral sampling, we divided the area into seven strata or sampling blocks with areas ranging from the 88 km^2^ (block D) to 165 km^2^ (block A). The forest cover ranged from the 15% in blocks A and E to the 49% in block B.

### 2.2 Data collection and analysis

From October 29th 2014 to May 9th 2015, we sampled the 900 km^2^ study area with 823 km of reconnaissance walks (recces) and transects. Since the population status and distribution of the Comoé chimpanzees was largely unknown at the beginning of this study, we first concentrated on localizing activity “hot spots” including nesting areas, tool use sites and promising places for camera trapping due to the presence of fresh signs of chimpanzee activity and tool use. In order to maximize the number of chimpanzee‐related observations, we conducted a stratified sampling program focusing on forested habitats (gallery forest and forest islands), although we also crossed long stretches of savanna (of the 823 km walked, 611 were in savanna) along which we recorded all chimpanzee observations.

**Figure 2:**
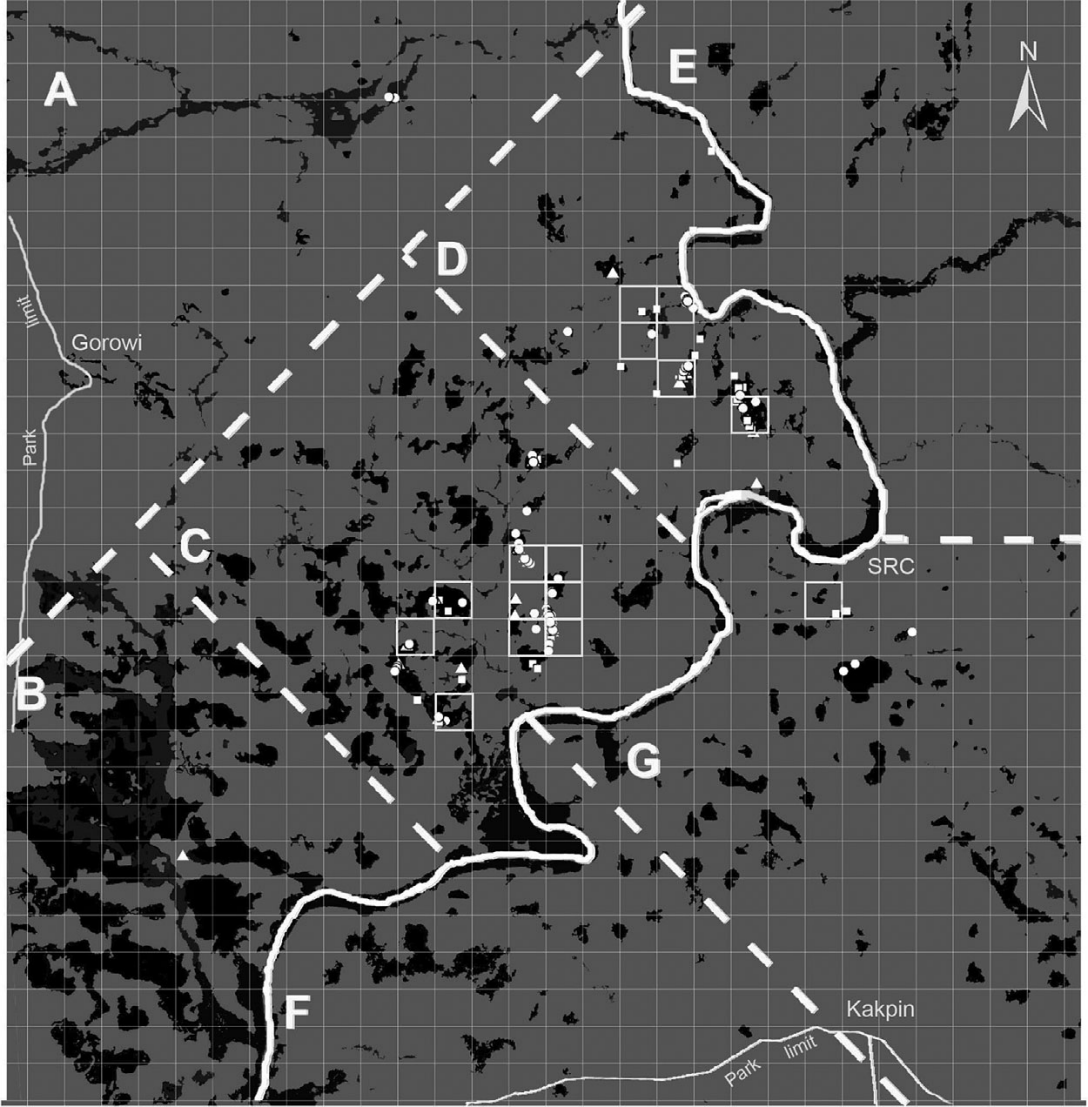
Distribution of fluid‐dipping sites found across the study area. The area covers 900 km^2^, divided into grid cells of 1 km^2^ each (white squares). Blocks C and D were the most intensively sampled, but we visited most of the forest patches in the study area. In the map, black and dark‐gray patches represent gallery forest or forest‐islands. The highlighted grid cells are where we recorded videos of chimpanzees. Triangles represent water dipping sites using only sponges, circles represent water‐dipping sites using sticks or sponges and squares represent honey‐dipping sites

Since this research was carried out within the framework of a larger project, which had as one of its main objectives to find chimpanzee communities suitable for long‐term study, we devoted more sampling time (80% vs. 20%) to the communities found in the strata closest to Research Station Comoé. Thus, we dedicated six weeks to sampling block C, 6 weeks to D, 2 weeks to E 2 weeks to G, and only 1 week to block A and one to B (See Figure 2). A team of three to four persons (the first author, two local assistants and from April to May a Master student) walked slowly (estimated walking speed of 1.5 km/hr) along reconnaissance routes and transects using a GPS Garmin 62st and a Suunto A‐10 compass. All tool‐use related observations were made ad libitum. We also recorded all direct and indirect observations of chimpanzee activity (i.e., feed, foraging, rest, social interact, travel, tool use), food sources (fruits, leaves, beehives, vertebrate, and invertebrate prey, drinking spots), habitat (gallery forest, forest island, woody savanna, bushy savanna, grass savanna), presence of mammals and evidence of human activity such as poaching, ilegal logging, old settlements, fishing, rangers patrols or tourism. We paid special attention to chimpanzee tool use sites (places where chimpanzees had been using tools, leaving the used tools behind). We recorded GPS coordinates and following standard etho‐archeological methods (Koops, McGrew, & Matsuzawa, 2013; McGrew et al., 2003; McGrew, Marchant, Hunt, & Hernandez‐Aguilar, 2007; Sommer et al., 2012), we photographed each tool in situ, measured the distances of the tools to the tree holes, distances of the plants used as tool‐source to the point of use, height and dimensions of the tree holes. We considered chimpanzee tools those sticks which presented clear signs of modification such as removed branches, leaves and sometimes bark and clear use‐wear signs such as blunt or frayed ends. Recently used stick tools (those that were not covered with mold, degraded, with parts missing, or partly eaten by termites) were collected and taken to the CRS for further analysis. We defined fluid‐dipping sites as tool use sites where we determined that either water or honey had been extracted with sticks. This was based on documenting fluid traces such as water, honey, wax, or mud (coming from the bottom of tree holes containing water), as well as the presence of bees or hive remains in tree holes, and whether the tools were found inserted or not into water or bee holes. Whenever possible, we inspected the content of the tree hole, measured its depth, height, and diameter and the presence/absence of water. In the laboratory, we labeled each tool, photographed and measured total length, brush tip length, and proximal and distal diameters. Using an experimental approach, we used a precision scale (accurate to 0.01 gm) to weigh the amount of water absorbed in 50 water dipping tools used by chimpanzees.

In November 2014, we set out 20 Bushnell HD trophy cams (model 119437), targeting mainly potential tool use sites, but also areas where it would be easy to identify individual chimpanzees, such as natural bridges. Cameras were placed using a systematic design across a grid of sampling cells of 1 km^2^, covering the areas with the highest concentration of nests and/or tool use sites, as well as cells in which we had made direct observations of chimpanzees, within the home‐ranges of three neighboring communities. Over the course of the study, we shifted 14 of the cameras within the same cell to focus on temporal activity “hot‐spots,” defined as places where tool use signs were very recent and abundant and moved six cameras between cells, following the seasonal movements of the chimpanzee groups. The cameras were programmed to record 60 sec videos, 24 hr/day, recording infrared videos when dark. One camera was lost to elephants a month before the end of the study.

In order to identify the individual chimpanzees in the videos, we used double blind recognition: two trained biologists (the first author and P. C. Köster) observed the videos independently. To further corroborate the accuracy of the identifications, we contrasted the results with observation of 50 sample videos by two untrained students. We tested the reliability of our identifications using Cohen's K coefficient to measure the percentage of coincidence between independent identification by the two trained biologists. For the tool‐use videos, we considered both episodes (defined as the sequence of fluid dipping, from the moment that one chimpanzee arrives at the fluid dipping site and starts the tool use, to the moment it leaves the site (Tonooka, 2001) and bouts (defined as each individual fluid dipping action, i.e., every time the chimpanzee introduced the tool in the cavity, extracted the fluid and consumed it) (Sanz & Morgan, 2007).

We carried out data analysis using the free software R‐Studio. We generated maps and carried out the spatial analysis using Quantum GIS. Since the data for tool length, tools per site and brush tip length did not follow a normal distribution, we used the Mann–Whitney test to look for significant differences between the means. We used Kruskal–Wallis test when testing differences between all tool types at a time.

We obtained estimations of community and party size from standing crop nest counts and camera trap videos and community and party composition from camera trap videos.

Data collection in the field was in compliance with the guidelines of the Ministere de l'Enseignement Superieure et de la Recherche Scientifique, adhered to the legal requirements of Ivory Coast, and the American Society of Primatologists principles for the ethical treatment of primates.

## 3 RESULTS

We found signs of permanent chimpanzee presence, including repeatedly used nesting and tool‐use sites, in five of the seven sampling blocks. In the other two blocks, we found some older scars left by chimpanzees on trees resulting from stone throwing behavior (Kühl et al., 2016), but no nest or stick tools. Based on estimates from nest counts on transects, we found larger chimpanzee communities in blocks A, C, and D than in B and G. Based on individual identification from camera trap videos, we found well structured chimpanzee communities composed of all sex/age classes in blocks C and D (we estimated a minimum of 54 and 20 weaned individuals, respectively, from standing crop nest counts). We estimated around 30 weaned chimpanzees in block A, 15 in block B and 10 in G. In blocks B and G, chimpanzee communities may have been smaller as a result of heavy poaching during the civil war that ended in 2011. As confirmed by the distribution of fresh nests and cells where videos were recorded, during the dry season, the most thoroughly studied chimpanzee communities in blocks C, D, and G moved from scattered forest islands after these had dried up to the gallery forests which retained pools of water for a longer period. Over the whole dry season, though, the chimpanzees continued visiting forest islands, to forage fruits, insects, and honey.

During the 6 month of sampling period, we found a total of 876 stick tools, including ant‐dipping tools, termite‐fishing tools, probes, and fluid dipping tools used both for honey and water. We also found 83 leaf‐sponges, as well as abundant stone tools. In this paper, we focus our analysis only on the fluid‐dipping tools, paying special attention to the water‐dipping tools.

We found water dipping sticks (WDT) in four of the sampling blocks (Figure 2 and Table 1), while we found honey dipping tools in three. These tools were always associated with tree holes containing either water or colonies of stingless bees (*Meliponini*). All the stingless bees found had their hives in tree holes and never in the ground or branch surfaces. Although we systematically checked all the trees containing honey‐bee (*Apis mellifera*) colonies, we never found any tool or signs of exploitation by chimpanzees.

**Table 1:** Number of fluid dipping tools found per sampling block

	A	B	C	D	G	Total
Honey‐dipping sticks (HDT)	0	0	137	107	45	289
Water‐dipping sticks (WDT)	7	0	198	70	8	283
Leaf‐sponges	0	7	59	17	0	83

A total of 283 water dipping sticks (WDT) were found at 77 water dipping sites, of which we confirmed revisits by the chimpanzees to 12. Two hundred and eighty nine honey gathering tools (HDT) were found at 38 different sites. Leaf‐sponges were found at 18 sites, six of which also included water dipping sticks. Sponges were found only in three of the sampling blocks, and in block B, only sponges and no water dipping sticks were found (it should be pointed out that in block B sampling effort lasted only 1 week). Sponges were found exclusively in or around those tree‐holes which were wide enough to allow the introduction of a chimpanzee's hand (diameter >8 cm). Stick tools, by contrast, were more frequently found associated with holes too narrow for a chimpanzee's hand (175 WDT in holes with a diameter range between 1.5 and 8 cm), although they were found in wider holes as well (108 WDT in holes with diameter between 8 and 34 cm). We never found sponges associated with honey hives. As we confirmed later with videos, chimpanzees used either sticks or sponges to dip for water from holes that were wide enough. Videos confirmed that sponges were produced using the well‐known techniques of chewing or folding the leaves in the mouth prior to use.

**Figure 3:**
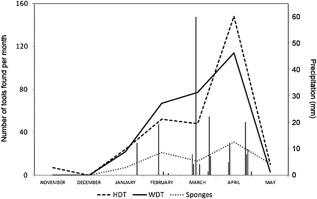
Number of fluid dipping tools found per month and precipitation. There was no precipitation between the end of November and the end of January. Precipitation (vertical bars in the graph) was recorded at the CRS, but we have no data for November, which had 6 days with rainfall. Sampling in May lasted only until the 9th. The peak in the use of honey dipping tools (HDT) may be related to the flowering of savanna trees, which is at its maximum in March, allowing meliponine bees to produce more honey in April

We found that among the 11 tree species that contained water dipping sites, 70% of the holes were in *Dialium guineense* and 12% were in *Cynometra megalophyla*. These also were among the most frequent species of the nine trees at which honey dipping tools were found (39% = *D. guineense*, 13% = *C. megalophyla*), although 16% of honey dipping sites were located in *Albizia adianthifolia* trees. Holes containing honey do not need to be as impermeable as those containing water, since bees are capable of sealing them with wax. Therefore, beehives can be built in trees were water could not otherwise be retained. Even though all the WDT and sponges were found during the second half of the dry season (from the end of January to May), when the small rivers were dry, there were still water pools remaining alongside riverbeds, and knuckle and footprints revealed that these were used by chimpanzees. Five of the water dipping sites were located alongside the large Comoé River, which always carried abundant water, and chimpanzees were seen drinking from this large river on one occasion. The use of WDTs peaked during the driest part of the season at the beginning of April, and was already in decline at the study's end at the beginning of the rainy season in May (Figure 3). Between the end of November 2014 and the end of January 2015, there was no precipitation, but small rivers in the core of the chimpanzee territory contained some water until January 15th. Despite the rainstorms in the late dry season, the pools present in the beds of small rivers continued shrinking due to evaporation and the repeated visits of large mammals; thus, by the end of April there were few and small pools in the riverbeds (only five in block C and three in block D). Trees, however, continued to have water in their holes, and thus provided an alternative source of water for the chimpanzees.

### 3.1 Tool characteristics

Both at water and honey dipping sites, we found that 26% (*N* = 152) of stick‐tools had been modified only minimally (the branches and leaves had been removed and the tips cut off). However, 74% of all fluid dipping stick‐tools (*N* = 620) were more extensively modified, with the bark removed and brush tips fashioned on one or both of the ends. As revealed by camera trap videos, chimpanzees produce brush tips by chewing the tip of the stick to loosen the fibers. Sixty‐three per cent of the HDTs had brush tips (*N* = 188) while 83% of the WDTs had them (*N* = 232).

**Figure 4:**
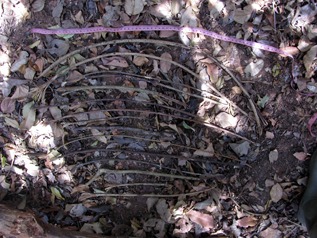
A tool assemblage (HDTs) representing a probable tool set. It was found in sampling block G at a honey dipping site which had been exploited earlier that day. All 15 tools were fresh, with remains and/or smell of honey and/or wax on their tips, but we do not know if they were used by one or more chimpanzees. The Meliponini beehive was in a fallen tree. We acquired at this site one video of a juvenile reusing a tool which had been left behind in the hive entrance. The second HDT from the top and the two at the bottom had brush tips of ordinary length and were considered honey collectors

Tool assemblages found at honey dipping sites were significantly larger than those found at water dipping sites, (6.44 ± SD 5.42 HDT per site, with a maximum of 19 tools per site for honey vs. 3.32 ± SD 3.55 WDT per site with maximum of 21 tools per site for water; *U* = 1002.5, *P* < 0.002). While honey assemblages appeared in most cases to be the product of a single episode (see Figure 4), videos confirmed that, in five of seven cases, water tools represented an accumulation of several days’ (2–5 days) episodes of tool use by multiple individuals. We found sticks without brush at 79% of the honey dipping sites (*N* = 38 sites, mean number of tools without brush per site = 2.7 ± SD 2.99 tools) but only in 32.47% of water dipping sites (*N* = 77 sites, mean number of tools without brush per site = 0.66 ± SD 1.35 tools), suggesting that this kind of tool was part of a tool set (two or more types of tools used sequentially to achieve a task) more probably in the case of honey dipping than water dipping.

Tools that we classified as honey‐perforators or probes, based on blunt or frayed tips and the absence of brush tip, were significantly longer than honey collectors, but not significantly thicker (Table 2). In contrast, the WDTs without brushes were shorter (although not significantly: *U* = 6341, *P* = 0.4224) than the ones with brushes. Based on three cases observed in the videos, it is likely that these later tools were selected by juveniles.

**Table 2:** Measurements of fluid dipping tools found

	Number of tools (N)	Total length (cm)	Proximal diameter (mm)	Brush‐tip length (cm)
Probable honey probe/perforator	101	59.34 ± 25.88	6.20 ± 1.97	—
Honey collector	188	56.76 ± 21.39*	5.92 ± 2.02	4.29 ± 2.9**
WDT without brush	51	48.42 ± 20.17	5.64 ± 2.02	—
Brush tipped WDT	232	51.06 ± 19.39*	5.59 ± 2.12	5.81 ± 4.67**

Mean total length ± SD, proximal diameter ± SD (measured at the most heavily worn tip) and Brush‐ tip length ± SD for the four types of stick tools that we found at honey and water dipping sites. WDT are water dipping sticks. Differences between tool types were tested with U Mann–Whitney test. Honey collectors were significantly longer than brushed tipped WDT (*U* = 17217, *P* < 0.0008)* and brush tips of honey collectors were significantly shorter than those of brushed tipped WDT (*U* = 24965.5, *P* < 0.0006)**.

Brush‐tipped HDTs (e.g., honey collectors) were significantly longer than brushed tipped WDTs (*U* = 17217, *P* < 0.0008). HDTs without brushes (e.g., honey probes) were longer than any of the other tool types (Kruskal–Wallis chi‐squared = 18.9641, *P* < 0.0003). This may be due to the bees choosing deeper holes as a defensive strategy, but we were unable to measure all of the beehive holes, since they were sometimes inaccessible to us. Beehives for which we could acquire accurate height measurements were significantly higher (*N* = 14, 6.27 ± SD 4.54 m) than water holes on trees (*N* = 32, 2.15 ± SD 2.73 m, *U* = 52, *P* < 0.00004). We found a weak positive correlation between water tree‐hole depth and WDT total length (*P* = 0.002, *R* ^2^ = 0.135). This may result from the fact that chimpanzees sometimes used long tools (*N* = 11) in shallow holes and short tools in deep wide holes (*N* = 13).

**Figure 5:**
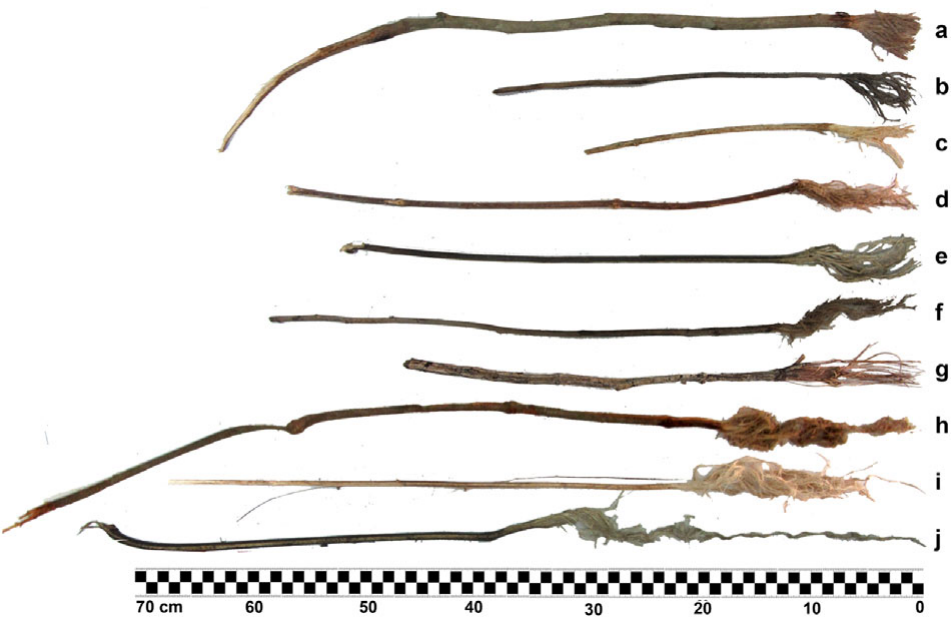
Sample of different water dipping sticks (WDT). The three WDT on top have ordinary brush tips, the four in the middle have long bruh tips and the three at the bottom have extremely long brushes with the fibers finelly detached, as example of what we called fiber‐sponges in the text. Tools a and h were used one after the other by adult male Hector in video 1 (see supplementary materials)

As we confirmed by camera trap videos and examination of the tools, the chimpanzees used the same technique to modify the tools with teeth or hands for both types of fluid dipping tools. This included; 1 Detaching the stick from a tree, vine or shrub with the hands; 2 Reducing the tool's length with hands and/or teeth, 3 Removing the leaves and branches with hands and teeth; 4 Producing a brush tip with the teeth. Some tools were further modified: 5 Removing completely the bark with hands and teeth; 6 Modifying the length after first use with hands and/or teeth; 7 Producing a second brush tip on the opposite end with teeth. The chimpanzees sometimes made an additional modification when producing especially long brushes (>5 cm long) by repeatedly chewing the tip during use (see Video 1 in Supplementary Materials). We found that this latter form of modification was more frequent in WDT (46% of brushes) than HDT (28% of brushes). Seventeen percent of the water dipping brushes were >10 cm long, with a maximum recorded length of 38 cm (see Figures 4 and 5).

WDT brushes (mean length of 5.81 ± SD 4.67 cm) were significantly longer than honey collector brushes (mean length of 4.29 ± SD 2.9 cm) *U* = 24965.5, *P* < 0.0006 (Table 2). In 51 cases (18% of all WDT brushes), chimpanzees produced a fiber‐sponge at the tip of the WDT by repeated chewing (see Figure 5).

### 3.2 Tool efficiency experiment

In order to test the relationship between brush length and water absorption, we introduced in the laboratory 50 WDTs, which had been used by chimpanzees, into a container full of water (depth 15 cm, width 10 cm) 10 WDTs were selected from each of five brush‐length categories (1st, No brush, 0 cm, 2nd = 0.1–2.5 cm, 3rd = 2.6–5.5 cm, 4th = 5.6–11 cm, 5th = 11.1–38 cm). After dipping, we weighed the water absorbed (see methods). We found a significant positive correlation (*R* ^2^ = 0.6001, *P* = 2.557 e^−11^) between the length of the brush and the amount of water absorbed.

**Figure 6:**
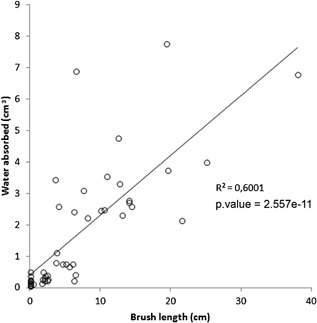
Correlation between brush‐tip length and water absorbed. Weight of water in grams is equivalent to volume in cm^3^ or milliliters. The dispersion of some points is due to differences in the diameter of the sticks: the bigger the diameter, the more water was collected

Fiber‐sponge brush tips were always longer and had finer and more detached fibers which made them more absorbent than ordinary brush tips (See Figure 3). A sample of 15 ordinary brush tips used in the absorption experiment (mean length 2.88 ± SD 1.36 cm) absorbed a mean of 0.77 ± SD 0.9 ml of water, while 15 fiber‐sponge tips (16.8 ± SD 7.5 cm) absorbed a mean of 3.68 ± SD 1.7 ml, five times more water per dip (*W* = 15.5, *P* < 9.6 e^−06^) (Figure 6).

### 3.3 Camera‐trap videos

**Figure 7:**
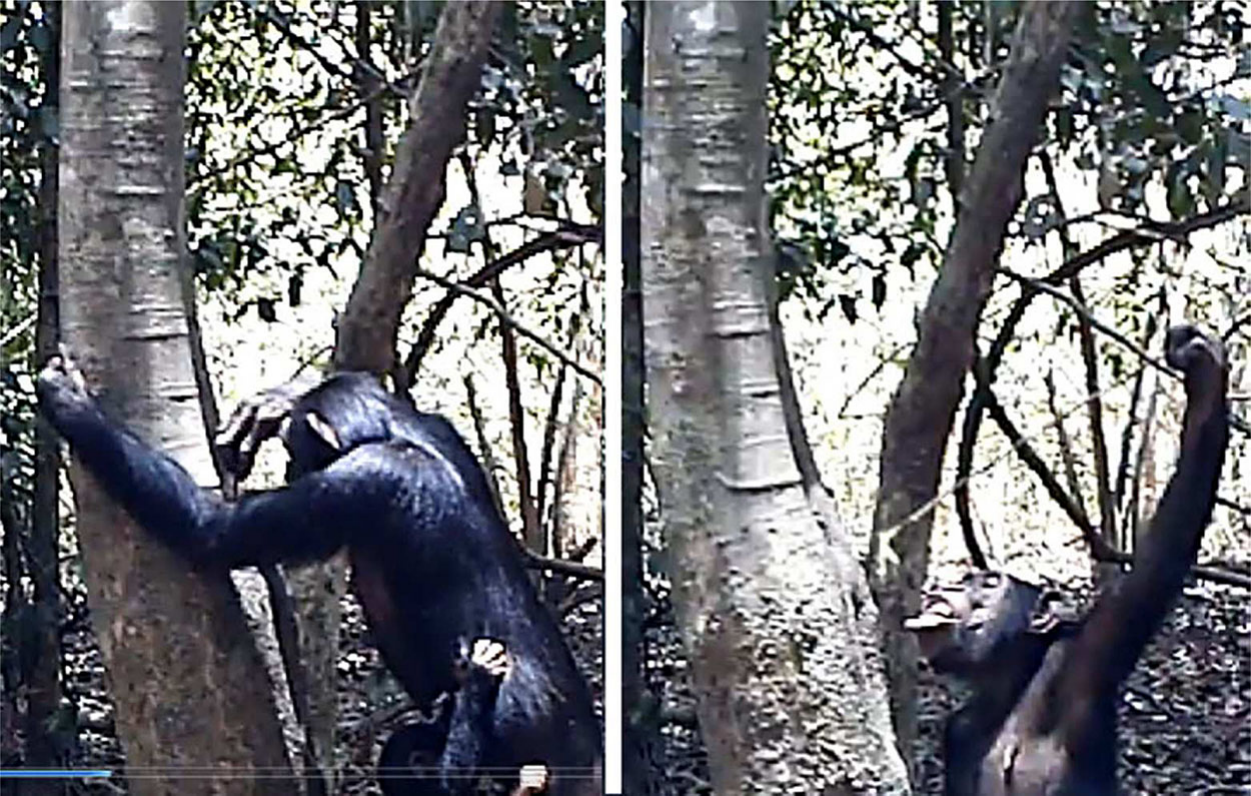
The adult female Cassandra drinks with a WDT from a tree hole. She introduces a WDT with her right hand. She retrieves it and holds it with left hand while drinking. Video capture, March 24 2015

We recorded 267 videos of chimpanzees using 20 cameras over a 6 month period, of which 110 were tool‐use videos. Seventy‐six of the tool use videos were of fluid dipping behavior, of which 39 included water dipping with sticks, three showed honey dipping with a stick, and the remainder (*N* = 34) were of leaf‐sponge use. This small number of honey‐dipping videos may be explained by the low revisit rate (we checked honey dipping sites periodically and only two were revisited, and on only one occasion, in more than 6 months of censusing) and the fact that the greater average height of honey sites (6.27 ± SD 4.54 m for beehives vs. 2.15 ± SD 2.73 m for water holes) complicated the installation of our cameras. In the sponge use videos, we recorded two adult males, six adult females, and five juveniles drinking with leaf‐sponges. We recorded one adult male, five adult females, one juvenile, and one infant using WDTs. The honey dipping videos were of one juvenile and two adult females (Figure 7).

In addition, we recorded 14 videos of chimpanzees checking waterholes in trees, presumably to see if there was any water left, with three adult males, five adult females, four juveniles, and three infants looking inside the holes or probing into them with discarded stick tools. We never observed in any of our videos a chimpanzee using its hands to gather water from tree holes, while four different monkey species were recorded doing so, baboon (*P. anubis*), Lowe's monkey (*C. lowei*), lesser spot‐nose monkey (*C. petaurista*), and white‐naped mangabey (*C. lunulatus*).

### 3.4 Drinking technique

**Figure 8:**
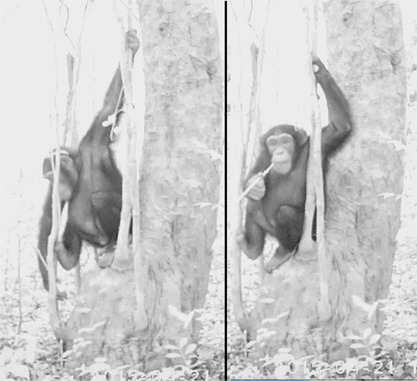
The juvenile male Laertes dips for water in a 1.5 cm wide hole at the base of a *D. guineense* tree. While drinking, he chewed the tip, finally producing a medium sized 4 cm long tip. He peeled all of the bark from his tool in order to make it fit in the narrow hole. Video capture, April 21 2015

Based on the videotapes (*N* = 38), we were able to determine that the technique used by the chimpanzees to drink using sponges did not differ from that reported for chimpanzees at other sites: leaves were collected from the immediate vicinity, then introduced in the mouth and chewed (73.5%) or folded (26.5%) to make them absorbent. Next, the chimpanzees dipped the sponge repeatedly into the water, taking it to the mouth, sucking it with the lips or pressing it between the lips and teeth. In three holes of depth exceeding 60 cm and diameter greater than 12 cm, the apes introduced their arm up to their shoulder into the hole to reach the water with the sponge. The water dipping technique included sticks which were always chewed before use, to prepare brush tips, and then repeatedly chewed producing long brushes or fiber‐sponges during the water dipping episode (*N* = 35). Sticks were torn off from saplings and fashioned by detaching branches, leaves, and bark while the chimpanzees were on the ground at the base of the tree hole. In four cases, videos revealed that tools were prepared at a distance (10–20 m) and transported to the site. In other 12 cases, we found remains of the tool preparation (detached leaves, broken saplings) up to 60 m away. We also found 25 tools that had not been detached from plants present within 10 m of the water dipping site. In 93 of the 111 water dipping bouts recorded on video, the tip was chewed after drinking and before reintroduction into the hole. The chimpanzees would keep the tools inside the tree holes for 2–10 sec, moving them slowly up and down to soak up the water. When repeated more than 10 times, the repeated chewing produced very long brushes or fiber‐sponges (see Video 1 in Supplementary Materials) (Figure 8).

## 4 DISCUSSION

**Figure 9:**
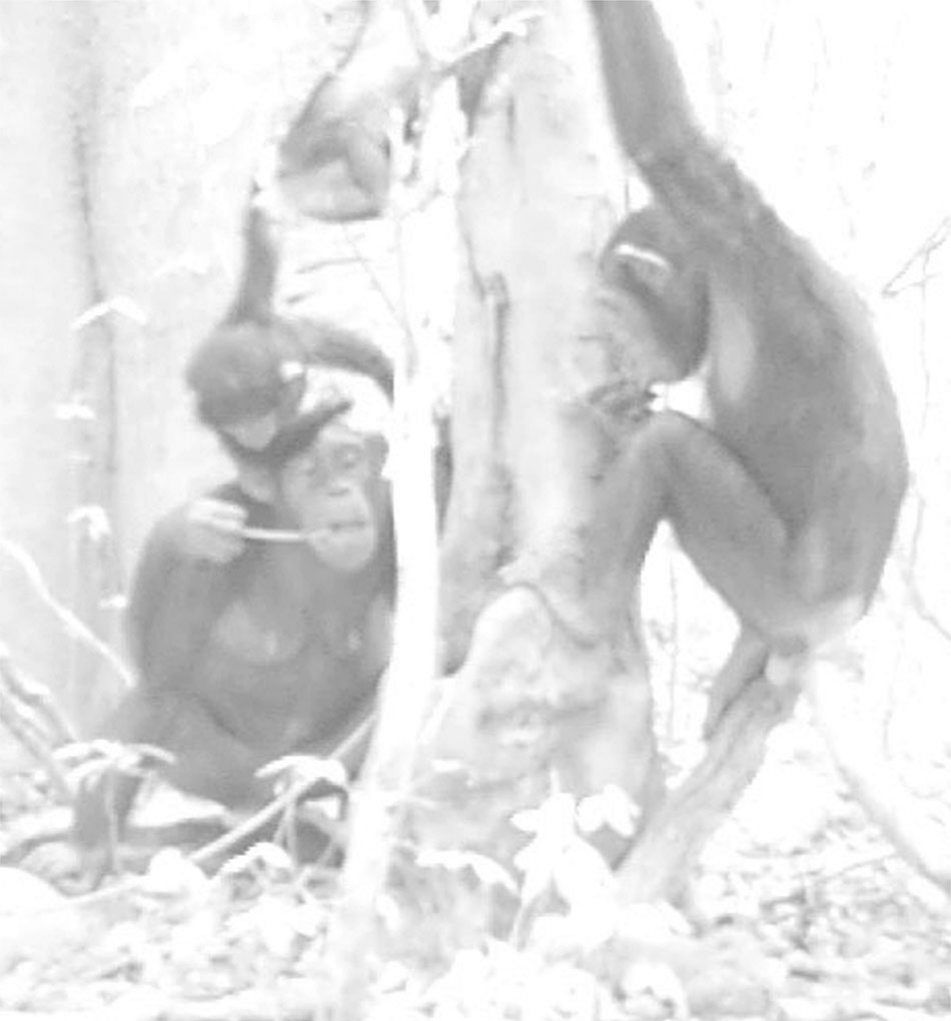
The adult female Andromaka drinks using a brush stick (WDT) that she carried already prepared to the site in her mouth, while the adult female Cassandra uses her sponge to drink at the same hole, which is wide enough to introduce her hand. The infants of both mothers observe the scene while playing. Video capture, April 25 2015

In this research, we examined tool‐use behavior in a population of savanna chimpanzees inhabiting a seasonally water restricted environment by testing several hypotheses related to tool dimension, manufacture technique, function, as well as whether tool‐use, and manufacturing techniques were limited to a particular community or common across several local populations. Previous studies indicate that savanna‐living chimpanzees modify their behavior, habitat use and territorial movements to adapt to water scarcity by moving in large mixed parties within their home ranges and concentrating around water sources during the dry season (Baldwin et al., 1982; Duvall, 2000, 2008; Hunt & McGrew, 2002; Pruetz & Bertolani, 2009; Tutin et al., 1983). Similarly, Comoé chimpanzees also moved within their ranges, as videos and fresh nests demonstrated, concentrating their activities during the dry season to forest patches that were within a radius of 2 km of the remaining pools in smaller rivers and used tools to obtain water and honey from tree holes during the late dry season. These chimpanzees were found to avoid the large river Comoé, where human activity (poaching, illegal fishing) is concentrated and where larger mammals such as monkeys, buffalos, antelopes, hogs, leopards, and hyenas go to drink. Unlike savanna chimpanzees both in Senegal (Galat et al., 2008) and Semliki‐Toro, Uganda (McGrew et al., 2007) who dig wells in the sand surrounding dirty pools of water, apparently to filter it, we never found signs of well digging at Comoé N. P., despite the fact that by April, the remaining pools were small and stagnant (Figure 9).

In this research, we tested the following hypotheses:

1. If Comoé chimpanzees develop tool‐use behaviors to confront their dry savanna environment then we expect to find evidence of adaptive tool‐use behavior produced by these chimpanzees particularly during periods of water scarcity.
  ‐ We found 283 stick tools (WDTs) used by the Comoé chimpanzees to obtain water from tree holes during the late dry season. 175 of these WDTs were used in tree holes too narrow for a chimpanzee's hand to be introduced, thus the tools allowed the chimpanzees to obtain water from otherwise inaccessible sources. The tools were found during the late dry season, when other water sources were scarce, which supports our hypothesis.
2. Assuming that tool technology in wild chimpanzees is learned by watching others, then we expect water dipping behavior to be common and widespread across the chimpanzee communities under study.
  ‐ The 283 WDTs were found distributed across four different sampling blocks that contain the home ranges of at least four different chimpanzee communities. We also recorded 39 videos of individuals of all sex/age classes using the WDTs. This evidence supports our hypothesis, suggesting that water dipping behavior with stick tools is common and widespread among Comoé chimpanzees.
3. Assuming that tools used by chimpanzees are specific to a particular task, we test the hypothesis that tools produced by Comoé chimpanzees to collect water (WDT) are different in dimensions and structure from those made to dip for and harvest honey (HDT).
  ‐ The mean total length of 289 HDT found during this study was significantly longer than the mean total length of 283 WDT (*U* = 17217, *P* < 0.0008). Sixty‐three per cent of HDT had brush tips, while 83% of WDT had them. Forty‐six per cent of WDTs had long brush tips (>5 cm) versus 28% of HDTs. Thus, WDTs were found to be different in dimensions and structure from HDTs, supporting our hypothesis.
4. Taking into account that chimpanzees produce tools with long brush‐tips to dip for water, we expect a direct correlation between brush length and amount of water absorbed.
  ‐ We carried out an experiment measuring the water absorbed by a sample of 50 WDTs and found that there is a significant correlation between brush length and water absorved (*R* ^2^ = 0.6001, *P* = 2.557e^−11^), thus, our hypothesis was supported by the result.
5. Considering that water is a less dense fluid than honey and longer brush tips are needed to absorb enough water per dip, we expect WDT brush‐tips to be longer than HDT ones.
  ‐ The mean brush length of the 188 brush‐tipped HDTs (4,29 ± 2,9 cm) was significantly shorter (*U* = 17217, *P* < 0.0008) than the mean brush length of 232 brush‐tipped WDTs (5,81 ± 4,67), thus supporting our hypothesis.

**Figure 10:**
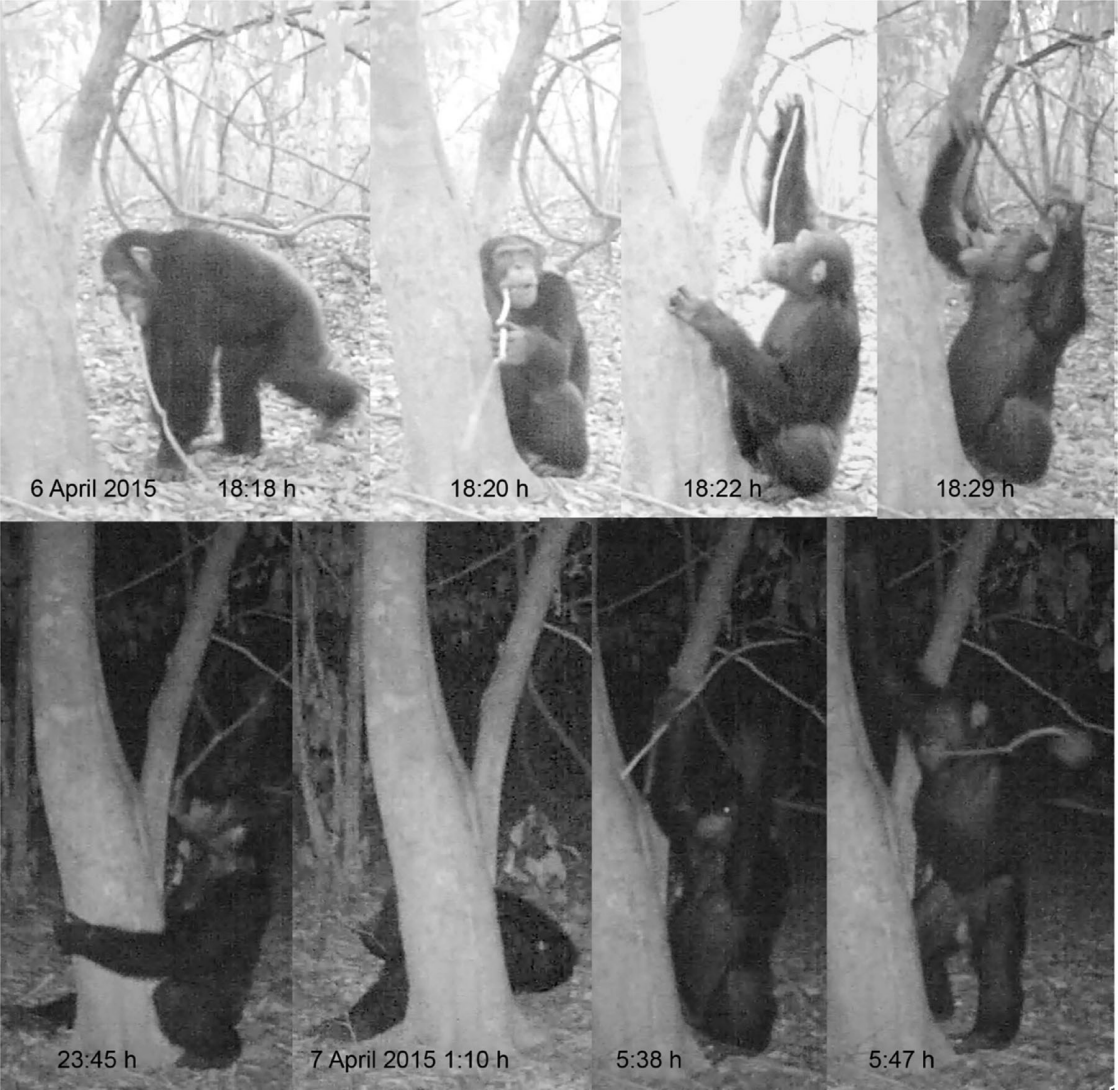
The adult male Hector brings WDT‐h after abandoning WDTa (see Figure 6). He chews the tip to produce a brush and then starts drinking. He introduces the tool mainly with his left hand, retrieving it with his right one, but sometimes switches hands. He stays drinking at the site for 12 hr, spending the whole night drinking and sleeping briefly beside the tree (6th frame). Nocturnal activity has been recorded in very few opportunities. Captures from Video 1 (see supplementary materials)

Sugiyama (1995) found in Bossou a similar seasonal pattern to the one found in our study, with the chimpanzees using sponges to drink from tree holes during the dry season. In other study sites, both savanna and forest chimpanzees drink from tree holes using moss, grass, leaf‐sponges or leaf spoons (Goodall, 1968; Hobaiter, Poisot, Zuberbühler, Hoppitt, & Gruber, 2014; Matsusaka et al., 2006; Sousa et al. 2009; Tonooka, 2001; Watts, 2008). These special drinking techniques appear to be adaptive solutions to conditions of reduced or unpredictable water availability during periods of the year or during dry years (Lanjouw, 2002). Clearly, the use of stick tools gives Comoé chimpanzees the ability to access water resources in narrow tree holes that would otherwise remain unavailable. The abundance at Comoé of *D. guineense* and *Cynometra megalophylla*, both tree species which produce abundant tree holes capable of retaining water, may have been a factor facilitating the development of the WDT technology. We do not know yet if these species are abundant in other study sites with similar habitats, future comparative research between sites could clarify the importance of this ecological factor (Figure 10).

The development of this behavior in Comoé may have been facilitated by the presence of rainstorms that fill the tree holes with water during the period of highest water scarcity, the late dry season. Matsusaka et al. (2006) also speculates about the possible role that climate change and reduced water availability could have played in the recent appearance of this behavior at Mahale, although in that case, only juveniles have shown the behavior in playing context, and they do not produce elaborated tools, but simple sticks.

We found that fluid dipping using sticks and sponges is common and widespread among the Comoé chimpanzees. While honey dipping with tools and drinking with leaf‐sponges are well known in chimpanzees, and are customary or habitual behaviors at many study sites across Africa (Bermejo and Illera, 1999; Bermejo et al., 1989; Boesch, Head, & Robbings, 2009; Dutton & Chapman, 2014; Gruber et al., 2009; Goodall, 1968; Matsusaka et al., 2006; Sanz & Morgan, 2007, 2009; Sugiyama, 1995; Watts, 2008; Whiten et al., 1999, 2001), water dipping with sticks is extremely rare and has only been described for juveniles in a play context at Mahale, not being neither customary nor habitual at this site, but of recent appearance (Matsusaka et al., 2006). Although Wrangham once observed a Mahale chimpanzee chewing a stem of *Aframomum* sp. to dip for water, this is not habitual or customary behavior at the site and he describes these tools as stem sponges (Whiten et al., 2001; Wrangham et al., 1994). Goodall (1968) described the use of grass leaves to dip for water at Gombe, but after repeated chewing, these grasses were used more like a sponge. Our findings at Comoé represent a more developed and frequent expression of the behavior. The brushed tipped WDTs produced at Comoé can, therefore, be considered a new variety of specialized tools, which is widespread in this population and adapted to a particular task. We have documented 77 water dipping sites and 283 WDTs distributed across the home ranges of at least four different chimpanzee communities. Based on 39 videos of chimpanzees of different age classes and sexes from neighboring communities dipping for water with sticks, we feel this represents a habitual or customary behavior in Comoé chimpanzees. Nevertheless, further research is needed to confirm this point.

### 4.1 Possible origin of the use of brush‐tipped sticks to drink

Sticks with brush tips are widely used by chimpanzees all across Africa, as honey collectors (Bermejo and Illera, 1999; Bermejo et al., 1989; Boesch et al., 2009; Dutton & Chapman, 2014; Gruber et al., 2009; Sanz & Morgan, 2007, 2009; Watts, 2008) and for the termite fishing in some Central African sites (Sanz & Morgan, 2007; Sugiyama, 1985). Comoé chimpanzees also proficiently and extensively use brush‐tipped honey collectors, we can therefore formulate the hypothesis of a probable extrapolation of the brush‐tipped sticks use from honey to water acquisition, that probably required little cognitive effort, since beehives and water holes often occur in the same trees species and chimpanzees frequently use probes to explore tree‐holes across their range.

Bossou chimpanzees solved the problem with the push and pull technique, retrieving leaf‐sponges with sticks from tree holes (Sugiyama, 1995). We cannot discard the possibility that Comoé chimpanzees may use the push and pull technique as well, because although we never recorded the behavior on video, we found sponges and sticks together at six tool sites.

It appears, however, that the extrapolation of honey dipping with brushed tipped collectors to water dipping is not that obvious for chimpanzees, since it has not been observed anywhere else, despite its clear utility, making water in narrow holes available, although the exact combination of ecological factors that could have favored the appearance of the behavior at Comoé could be absent in other sites.

At the Goualogo and Loango research sites in Central Africa, the chimpanzees have been observed to use tool sets for honey gathering, with the following sequence of tools: 1. Thick sticks as pounders to open the hard protective wall of the beehives; 2. Enlargers to make holes wider; 3. Probes to measure the depth of the honey; and 4. Collectors with brush tips to gather the honey (Boesch et al., 2009; Brewer & McGrew, 1990; Sanz & Morgan, 2009).

Given that Meliponini beehives in Comoé N. P. are only protected by thin wax walls, they are easy for the chimpanzees to perforate (personal observation). We suggest, therefore, that many of the honey tools without brush tips could have been used as perforators or probes whereas the tools with brush tips were collectors. If this is correct, then most of the groups of tools found at honey gathering sites were probably tool sets (i.e., assemblages of several different types of tools used one after the other to achieve a goal), as most of them had been found freshly used probably following a single episode of honey dipping (although we do not know if by a single individual or several at the same time).

We have found that the WDTs produced by Comoé chimpanzees are significantly different in dimensions and absorption capacity from HDTs, which indicates specialization. Further research is needed to determine if the production of brush‐tipped tools, the extrapolation of their use to different contexts and the additional improvement of longer brush‐tipped fiber sponges qualifies for a case of cumulative culture.

Comoé chimpanzees have successfully tackled the challenging environmental conditions of their harsh habitat not only by generalizing the use of brush sticks to both honey and water, but developing a new variety of brush tool with a specially modified tip which increases the effectiveness of the tool. It appears to be habitual or customary at Comoé, observed in all age and sex classes, although we need further research to confirm it. We consider the water dipping with brush‐tipped WDT behavior of the Comoé chimpanzees as a new type of tool‐use behavior.

From all the data collected up to now we cannot yet conclude if making and using WDTs to drink water is more a choice than an ecological need, habituating the chimpanzees and evaluating the relative importance of water consumption from the different sources will allow us to answer this question in the future. The water‐acquisition technology of Comoé chimpanzees represents a new set of chimpanzee traditions. Our study demonstrates the importance of carrying out detailed studies of threatened and isolated chimpanzee populations. Without these kind of studies, we will lose forever the opportunity to discover fascinating cultural variations and unique adaptations that could provide with important clues to better understand human evolution. Tool use gives chimpanzees a clear advantage in water acquisition with respect to any other sympatric animals. Exclusive access to key resources through tool use could also have spurred increased rates of cultural evolution in our own ancestors. We must preserve and study the Comoé chimpanzees not only because they can provide us with important clues about our evolution, but also because they probably are the second largest population in Ivory Coast and the only known viable population of savanna chimpanzees remaining in the country. We are just scratching the surface in our understanding of this population with its extraordinary behavioral and cultural richness.

## Supporting information

Additional Supporting Information may be found online in the supporting information tab for this article.

Supporting Information Video 1.
